# Publisher Correction: Water training initiates spatially regulated microstructures with competitive mechanics in hydroadaptive polymers

**DOI:** 10.1038/s41467-024-52330-5

**Published:** 2024-09-23

**Authors:** Wenbo Chen, Caoxing Huang, Philip Biehl, Kai Zhang

**Affiliations:** 1 Sustainable Materials and Chemistry, Department of Wood Technology and Wood-based Composites, University of Göttingen, Büsgenweg 4, D-37077 Göttingen, Germany; 2 Jiangsu Co-Innovation Center for Efficient Processing and Utilization of Forest Resources, College of Chemical Engineering, Nanjing Forestry University, 210037 Nanjing, China

**Keywords:** Mechanical properties, Polymers, Structure of solids and liquids

Correction to: *Nature Communications* 10.1038/s41467-024-50328-7, published online 19 July 2024

In Fig. 2b of this article the label ‘Bulk Mechano-responsiveness’ incorrectly read ‘Bulk Mechan responsiveness’. The original article has been updated.

