# Supplementary material for: Water training initiates spatially regulated microstructures with competitive mechanics in hydroadaptive polymers
Source: Nat Commun. 2024 Jul 19;15:6093. doi: 10.1038/s41467-024-50328-7 (PMC11271527; doi:10.1038/s41467-024-50328-7)
Supplement: Supplementary file 1 — Supplementary Information [file 41467_2024_50328_MOESM1_ESM.pdf]

Supplementary Information

Water training initiates spatially regulated microstructures with competitive mechanics in hydroadaptive polymers

Wenbo Chen[1], *Caoxing Huang*[1,2], *Philip Biehl*[1], *Kai Zhang*[1*]

Address:

[1] Sustainable Materials and Chemistry, Department of Wood Technology and Wood-based Composites, University of Göttingen, Büsgenweg 4, D-37077 Göttingen, Germany.

[2] Jiangsu Co-Innovation Center for Efficient Processing and Utilization of Forest Resources, College of Chemical Engineering, Nanjing Forestry University, 210037 Nanjing, China.

*Corresponding author. Email: kai.zhang@uni-goettingen.de

The PDF file includes:

Supplementary Note 1-12
Supplementary Fig. 1-17
Supplementary Table 1
Supplementary References

Supplementary Note

1. Nuclear magnetic resonance (NMR) analysis

Signals from both cellulose backbone and phenoxyacetate groups were observed from liquid-state ^{1}H and ^{13}C NMR spectra. Within the ^{1}H NMR spectra (Supplementary Fig. 1A), the signals from 6.9 to 7.4 ppm represent the hydrogen at β position of carboxyl groups and the hydrogen from the benzene ring[1,2]. The peaks between 3.0 to 5.8 ppm originate from cellulose backbone[3]. Within the ^{13}C NMR spectra (Supplementary Fig. 1B), the peak at 169 ppm denotes the carboxyl groups, while the signal at around 158 ppm represents the substituted carbons of the benzene ring on phenoxyacetate groups and the signal at around 115 ppm originates from the carbon at α position. The signals from 126 to 135 ppm correspond to the carbon from benzene ring, while the signals from 60 to 105 ppm are derived from cellulose backbone[3]. Moreover, C_6 of the anhydroglucose units of cellulose has a new signal at ~63 ppm in addition to the signal at ~60 ppm, showing the esterification at C_6 position. In comparison, the signal attributed to C_1 at ~103 ppm is not split, indicating no modification of C_2 position.

2. Elemental analysis

The degree of substitution (*DS*) of CPaE was calculated according to previously published method based on the content of carbon with modification[4]:

$$DS = \frac{162.14 \times c - 72.066}{96.088 - 118.135 \times c}$$

Where c is the content of elemental carbon.

The carbon content of CPaE was measured using elemental analysis to be averagely 50.1%.

3. Static mechanical properties

The as-prepared mid-CPaE membranes were strong, and tough with a tensile strength of 92.9 ± 3.1 MPa, Young's modulus of 1.8 ± 0.1 GPa, elongation at break of $9.2 \pm 0.7\%$ and fracture work of 5.0 ± 0.8 MJ m^{-3} (Supplementary Fig. 3A). The numerous entanglements among CPaE polymer chains as well as other interactions, including hydrogen bonds and π-π stacking should also contribute to its high strength and toughness.

The hydrated thick-CPaE with the thickness of 48-50 μm showed trivial linear elastic behavior compared to hydrated thin-CPaE of 4-6 μm (Supplementary Fig. 7). The tensile

strength, Young's modulus, elongation at break and fracture work of (Supplementary Fig. 7A) hydrated thin-CPaE membrane was 83.8 ± 3.8 MPa, 2.3 ± 0.1 GPa, 8.0 ± 0.7% and 4.7 ± 0.5 MJ m^{-3}, while (Supplementary Fig. 7B) hydrated thick-CPaE membrane was 63.6 ± 1.8 MPa, 1.0 ± 0.1 GPa, 21.6 ± 2.8% and 10.1 ± 1.6 MJ m^{-3}, respectively. This should be attributed to varied entanglement density of CPaE membranes with distinct thickness, resulted in a harder or softer elasticity in response to the applied stress.

4. Recyclability of CPaE membranes and the static mechanical properties after 10, and 20 times of regeneration

The recyclability of CPaE membranes were verified by repeated dissolve the membranes in DMF without heating, and then dried to from membranes. Static mechanical properties of mid-CPaE membranes strips after 10, 20 times of regeneration were analysed (Supplementary Fig. 8). These membrane strips after regenerated for 10 times were strong, stiff and tough with tensile strength of 82.0 ± 0.9 MPa, Young's modulus of 1.8 ± 0.1 GPa, elongation at break of 10.6 ± 2.3% and fracture work of 6.1 ± 0.1 MJ m^{-3}, while tensile strength of 94.4 ± 1.6 MPa, Young's modulus of 1.8 ± 0.1 GPa, elongation at break of 8.4 ± 0.3% and fracture work of 5.2 ± 0.3 MJ m^{-3} after regenerated for 20 times. Although being regenerated above 20 times, the mechanical properties of CPaE membranes strips remain constant to those before regeneration, showed an impressive flexibility and durability. This demonstrated that CPaE could be efficiently recycled and reused at ambient environment.

5. Dynamic Mechanical Thermal Analysis (DMTA) results

The creep behavior of hydrated CPaE membranes at 25 °C and 95% RH provided information about the variable deformation (Supplementary Fig. 12A). After 2 hours, the deformation of the hydrated mid-CPaE showed stabilized at 3.9%, whereas the hydrated thin, and thick-CPaE showed much larger at 9.0 and 6.9%, respectively.

Additionally, a brittle fracture of as-prepared mid-CPaE membranes occurred at -80 °C and no substantial changes of the mechanical properties in the alternatively established temperature scans ranging from 5 to 90 °C were observed (Supplementary Fig. 12B).

In comparison, RH hysteresis tests were conducted by using as-prepared and hydrated thin, and thick-CPaE (Supplementary Fig. 12C to F). The storage modulus of as-prepared thick-CPaE

decreased from 4.75 ± 0.04 GPa to 3.82 ± 0.03 GPa at 18 ± 2% RH during a RH cycle (Supplementary Fig. 9C). Correspondingly, the damping factor rose from 0.066 ± 0.001 to 0.085 ± 0.004. Additionally, the storage modulus of hydrated thick-CPaE increased from 2.28 ± 0.04 GPa to 2.74 ± 0.08 GPa at 92 ± 2% RH during a RH cycle (Supplementary Fig. 12D). Correspondingly, the damping factor remained constant at 0.101 ± 0.002. By contrast, the storage modulus of as-prepared thin-CPaE gently decreased from 5.40 ± 0.16 GPa to 5.26 ± 0.06 GPa at 18 ± 2% RH during a RH cycle (Supplementary Fig. 12E). Correspondingly, the damping factor slightly increased from 0.080 ± 0.002 to 0.085 ± 0.004. Additionally, the storage modulus of hydrated thin-CPaE increased from 1.80 ± 0.08 GPa to 2.08 ± 0.04 GPa at 92 ± 2% RH during a RH cycle (Supplementary Fig. 12F). Correspondingly, the damping factor slightly decreased from 0.145 ± 0.003 to 0.132 ± 0.008.

The critical effect of water in modulating the competitive plasticity and viscoelasticity of CPaE membranes were verified by performing temperature-frequency-humidity dynamic mechanical thermal analysis (T-f-RH DMTA). As shown in Supplementary Fig. 13A, the storage modulus of CPaE membranes under 90 °C and 30% RH decreased steadily (17.61%) from 2.84 GPa ± 0.10 GPa to 2.34 ± 0.05 GPa with decreasing frequency from 100 Hz to 0.1 Hz. Correspondingly, the damping factors increased gently (13.48%) from 0.089 ± 0.005 to 0.101 ± 0.002. In comparison, the storage modulus of CPaE membranes under 30 °C and 92% RH decreased sharply (30.89%) from 1.91 ± 0.08 GPa to 1.32 ± 0.09 GPa with decreasing frequency from 100 Hz to 0.1 Hz. Correspondingly, the damping factors increased abruptly (30.52%) from 0.154 ± 0.001 to 0.201 ± 0.008. In addition, T-f-RH DMTA was carried out under low temperatures and diverse RH level (Supplementary Fig. 13B). Obviously, substantial changes of storage modulus and damping factors for CPaE membranes were observed only at very high RH level, for example at 91% RH, while remaining basically unchanged at other RH levels. Moreover, the same phenomenon was also observed in the loss modulus (Supplementary Fig. 13C to D).

Both the as-prepared and hydrated CPaE exhibited prominent stability after 4 hours of time sweep tests at 25 °C, 10 Hz (Supplementary Fig. 14). The storage and loss modulus of as-prepared CPaE at 10% RH showed 5.06 ± 0.19 GPa and 0.33 ± 0.05 GPa, while hydrated CPaE at 95% RH showed 2.01 ± 0.05 GPa and 0.23 ± 0.01 GPa, respectively.

6. Calculation of hydrogen bonding energy between CPaE and water

Optimized atomic configuration of minimum point on the potential energy surface of CPaE-H_2O showed the hydrogen bonds pattern and binding sites between CPaE and H_2O (Supplementary Fig. 17). The single H-bonds occurred between water and the carbonyl group, while the double H-bonds occurred between water and -OH group on C_2 and C_3 of the glucopyranose, oxygen (O) atom on the glucopyranose and phenoxyacetyl group, O atom on the glucopyranose and the glycosidic linker, respectively.

The hydrogen bonds energy was calculated using the B3LYP generalized function with DFT-D3 (*BJ*) dispersion correction, and the wave function was calculated by combining the ma-TZVPP basis set, then analyzing the bond critical point electron density and fitting with the following formula[5]:

$$\Delta E \approx -223.08 \times \rho(r_{BCP}) + 0.7429$$

The calculated results of electron density and hydrogen bonds energy between CPaE and water were shown in Supplementary Table. 1.

7. Description and investigation of the water-responsive dual-mechanic functionalities

The bulk and differential mechano-responsiveness of materials represented their water-responsive dual-mechanic functionalities. Particularly, the differential mechano-responsiveness was explained by the mechanical features of the hierarchical microstructure with inner elasticity support area and outer plasticity adaptation area. Moreover, we found that this characteristic also could be observed in cellulose derivatives with other substituents and low degree of substitution (DS) (e.g. 0.2-0.4), such as cellulose benzoate (Supplementary Fig. 4C). Nevertheless, such characteristic did not appear as the DS was further increased to 1) range of 0.8-1.0, where the water-associated mechanical properties were similar to that of those with lower DS (Supplementary Fig. 6A to C); 2) 1.5 and above, where the affection of water on mechanical properties were negligible (Supplementary Fig. 6D to F). These findings suggest that 1) the intrinsic molecular basis of CPaE polymers, characterized by extensive entanglements and strong noncovalent effects, including the inter/intramolecular hydrogen bonds; 2) the subtle balance between hydrophilicity and hydrophobicity of CPaE polymers, have a substantial impact on the observed behavior.

8. Experimental demonstration of thin and thick-CPaE via water training strategy

The water-responsive dual-mechanic functionalities of thin, and thick-CPaE membrane strips were examined via water training and subsequently air training strategies (Supplementary Fig. 5). Water shapes can be generated in a much shorter time (demonstrated as 2 hours) for the thin-CPaE membrane strips (4-6 μm) (Supplementary Fig. 5A), while thick-CPaE membrane strips (46-52 μm) take longer (demonstrated as 120 hours) (Supplementary Fig. 5B to C). However, none of them allows for the subsequent shape recovery in water. This is the result of irreconcilable situation between the development of plasticity and the distinct viscoelasticity properties of each material. Thus, they are not suitable to be used as hydroadaptive polymers.

9. Supplementary Description of the modified viscoelastic-plastic model

As shown in Supplementary Fig. 11, the dashpot with low viscosity does not present much resistance to the deformation when mid-CPaE membranes were immersion in water with low applied force, resulting in a dominated elastic energy storage in the equilibrium spring of the outer layer. With accelerated stress relaxation induced by plasticity, the equilibrium spring would move the dashpot back to its original position while the delayed elastic deformation occurred in the non-equilibrium spring of the inner layer. Upon unloading, new deformation generally occurred due to the requirement of stress balance, resulting in an elastic energy redistribution among the two equilibrium and non-equilibrium springs. Nevertheless, the deformation in the viscoelastic branch was negligible because of the much higher modulus of non-equilibrium spring compared to equilibrium spring, which causes the regeneration of delayed elastic deformation introduced in water. After drying the membranes in air, the elastic energy between the two springs was redistributed again. As cycling the membranes in air and water, the force in the equilibrium spring should drive the non-equilibrium spring back to the original location due to the viscosity transition, followed by shape recovery.

10. ATR-FTIR spectra of the CPaE membranes soaked in D_2O for 5 min

As for the CPaE membranes soaked in D_2O for 5 min (Supplementary Fig. 15), the intensity of *v*(OH) and *v*(OD) bands strongly increased. Then, during the evaporation, both bands continuously decreased with *v*(OH) band almost to its initial intensity as before soaking. At the

same time, ν(OD) band still remains partially, indicating the presence of a small amount of D_2O within the CPaE polymer network.

11. Analysis on Polarized Optical Microscopy (POM) images for air-dryed CPaE membranes

The air-dried CPaE membranes were trained in water for 3 min and 3 hours before the observation using Polarized Optical Microscopy (POM) (Supplementary Fig. 9). The cross section of the two specimens look identical and are transparent under natural light. Under polarized light, however, the specimen (with 3 hours water training) becomes colored because of its differentiated stress distribution in the inner and outer layers, while the specimen (with 3 min water training) completely merges into the black background.

12. Time-dependent water sorption dynamics in CPaE membranes

To understand the water sorption dynamics in CPaE membranes, time-dependent water sorption curve was recorded (Supplementary Fig. 10). The adsorbed water content increased from 0.084 ± 0.010 to 0.095 ± 0.014 as the immersion time increasing from 5 min to 3 hours, and remained constant thereafter.

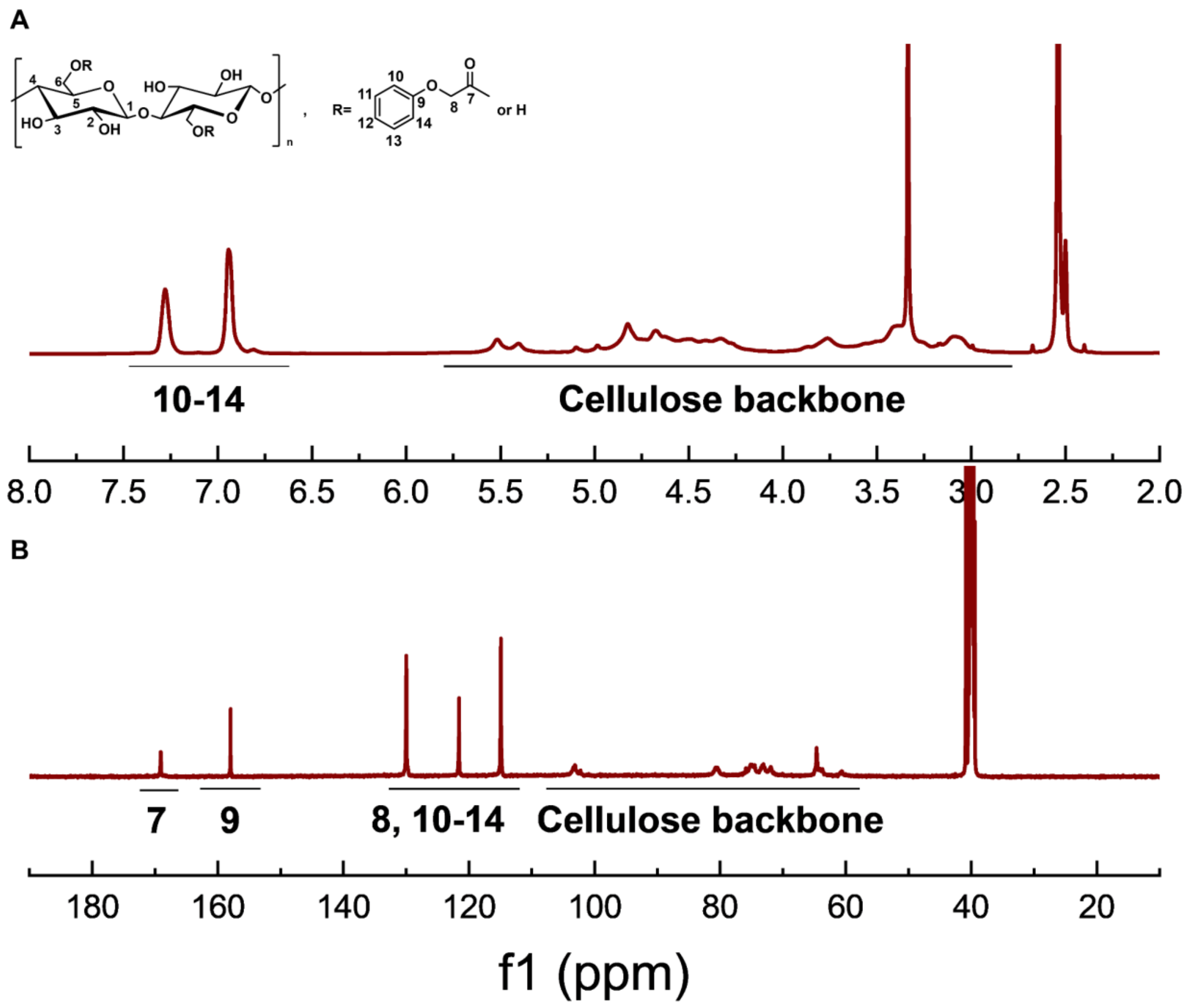

Supplementary Fig. 1.

(A) ^{1}H and (B) ^{13}C NMR spectra of cellulose phenoxyacetate (CPaE) in DMSO-d6.

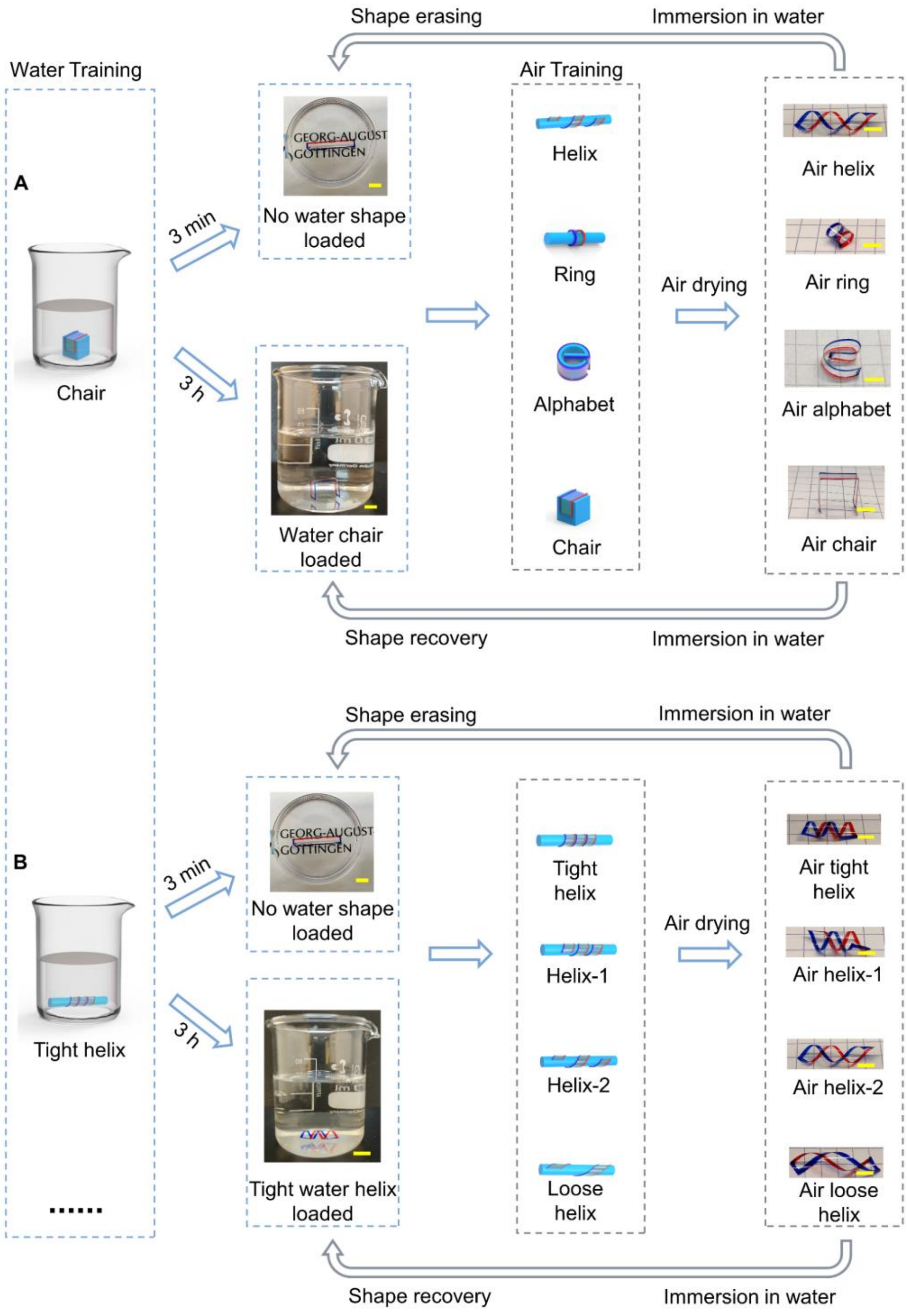

Supplementary Fig. 2.

A to B, Experimental demonstration of loading (A) water chair and (B) water helix with distinct pitch into the mid-CPaE membrane strips via water training strategy, and their water-responsive dual-mechanic functionalities. Scale bars, 5 mm.

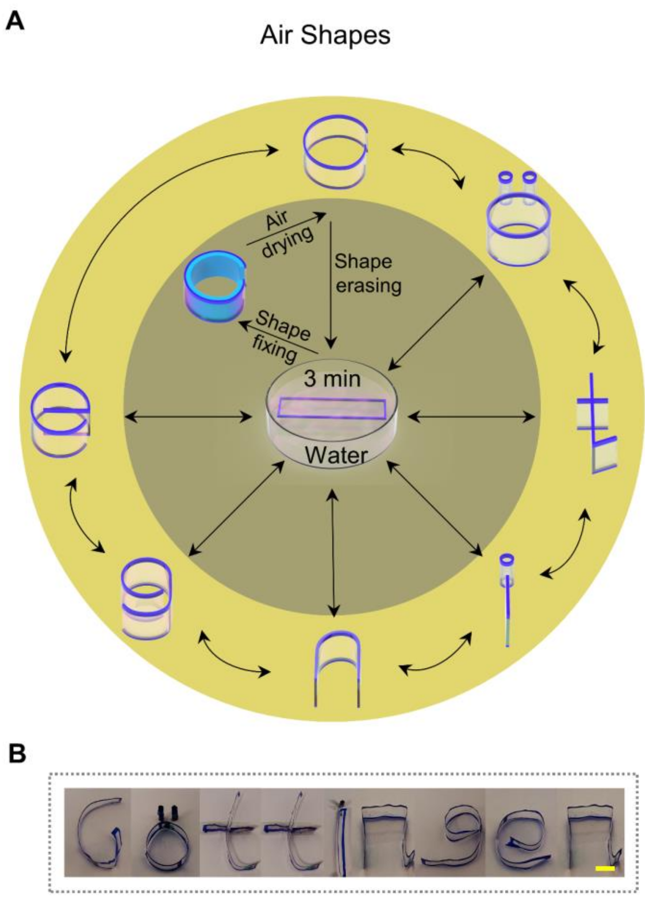

Supplementary Fig. 3.

A, Schematic flow charts showing the reversible process for continuously training the same CPaE membrane strip into versatile shapes (for example alphabets) via the water training strategy for 3 min to fix air shapes, followed by erasing the shapes in water. B, A set of digital images showing the motif of Göttingen. Scale bars, 5 mm.

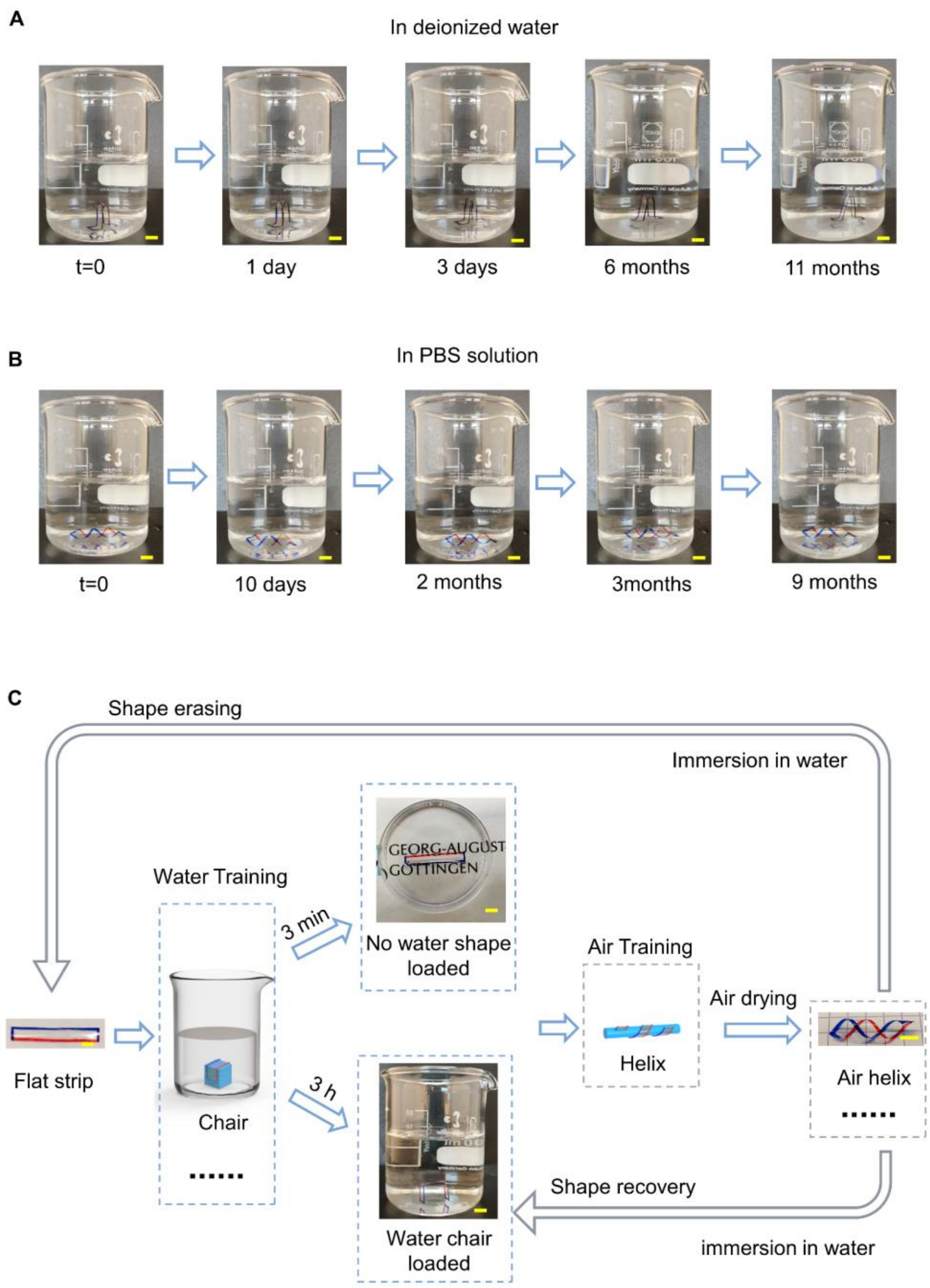

Supplementary Fig. 4.

A-B, Shape stability of CPaE membranes with loaded water shape stored in (A) deionized water, and (B) PBS solution. (C) Experimental demonstration of loading water chair into CBE membrane strips (supplementary cellulose derivative with low DS) via water training strategy, and their water-responsive dual-mechanic functionalities. Scale bars, 5 mm.

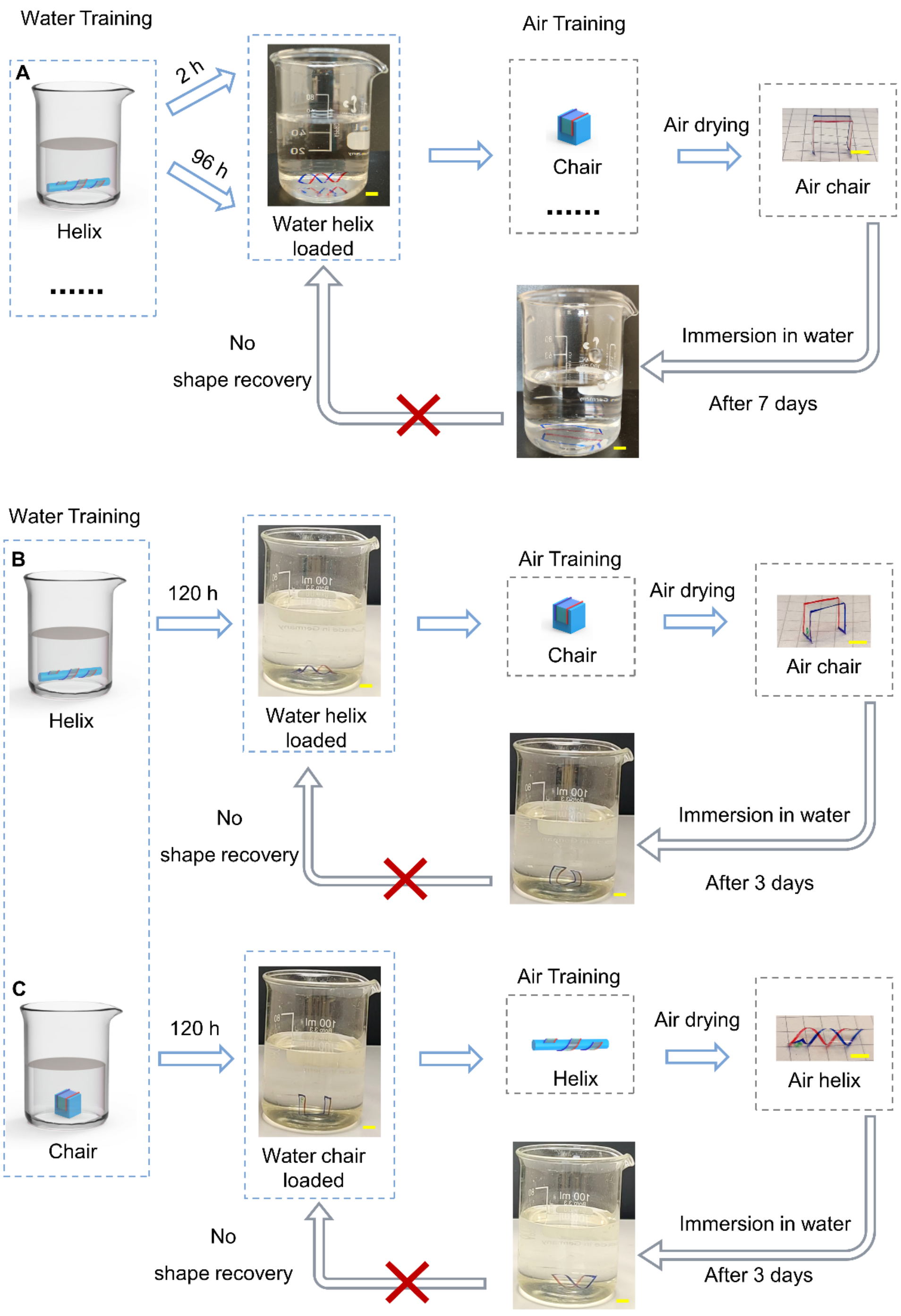

Supplementary Fig. 5.

A to C: Experimental demonstration of loading water shapes into (A) thin-CPaE membrane strips, (B to C) thick-CPaE membrane strips via water training strategy, and their invalid water-responsive dual-mechanic functionalities. Scale bars, 5 mm.

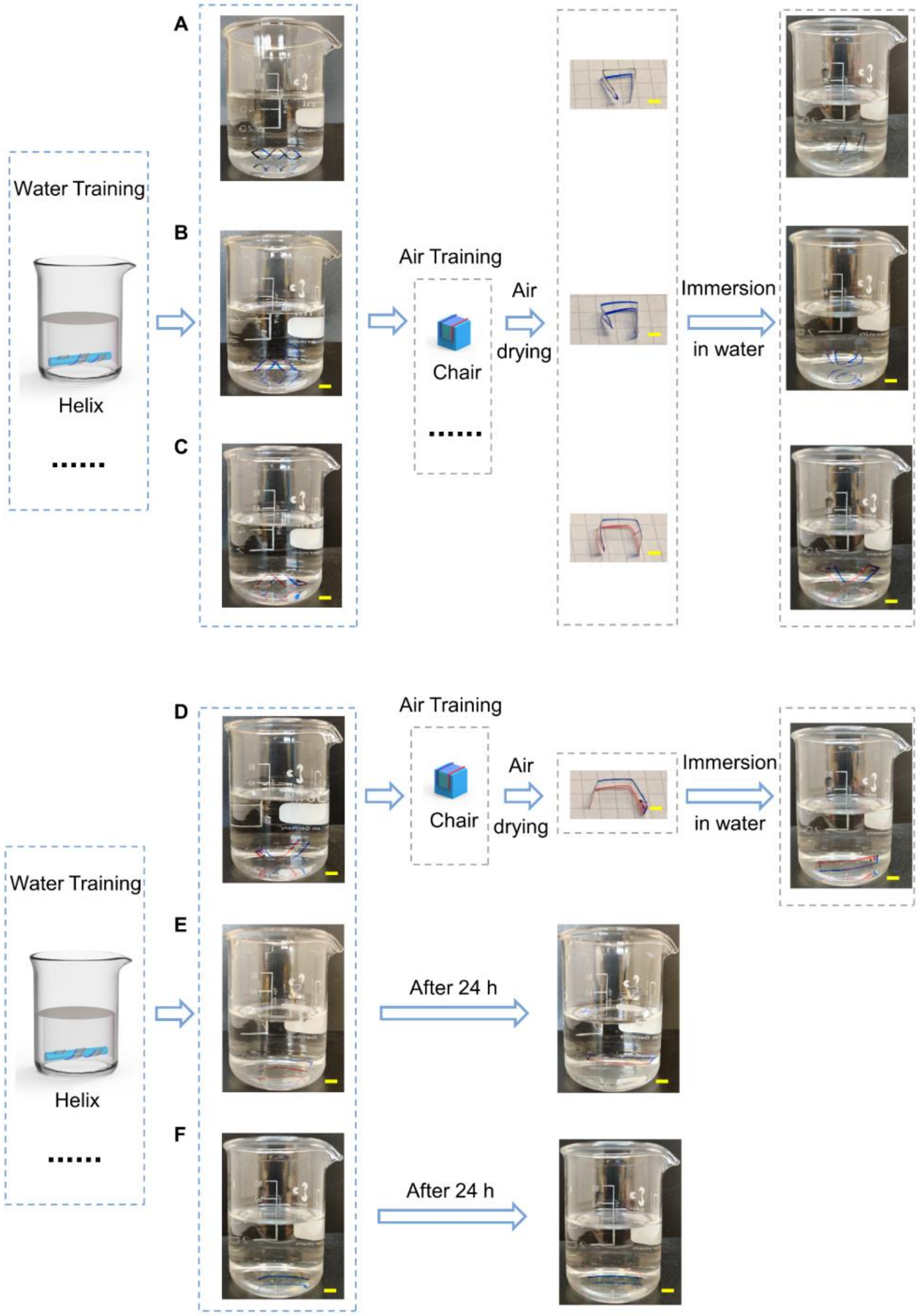

Supplementary Fig. 6.

A-F, Experimental demonstration of loading water helix into the (A) $CBE_{0.85}$, (B) $CPaE_{0.86}$, (C) $CSE_{1.0}$, (D) $CUE_{1.49}$, (E) $CUE_{2.35}$, (F) $CSE_{2.24}$ via water training strategy, and their invalid water-responsive dual-mechanic functionalities. Scale bars, 5 mm.

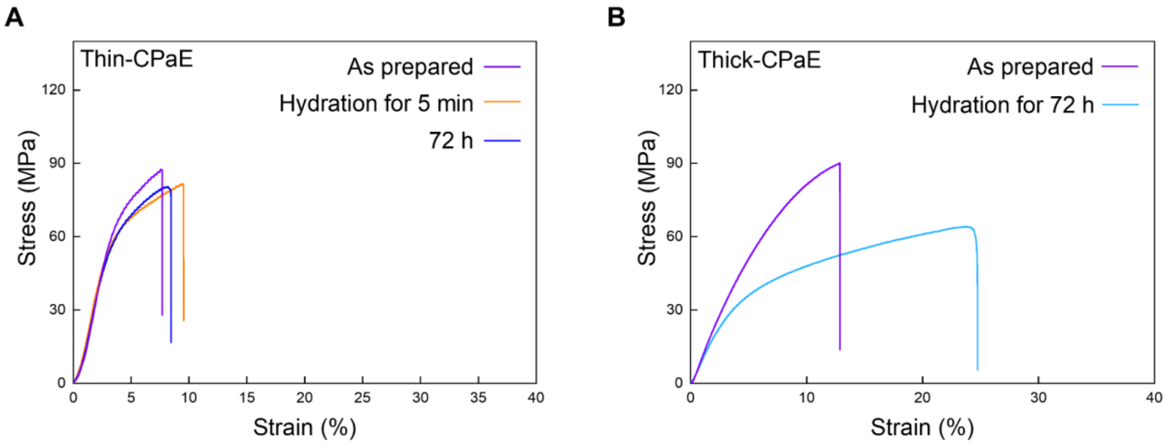

Supplementary Fig. 7.

Stress–strain curves of thin, and thick-CPaE membranes. (A) as-prepared thin-CPaE and after hydration for 5 min and 24 hours. (B) as-prepared thick-CPaE and after hydrated for 72 hours.

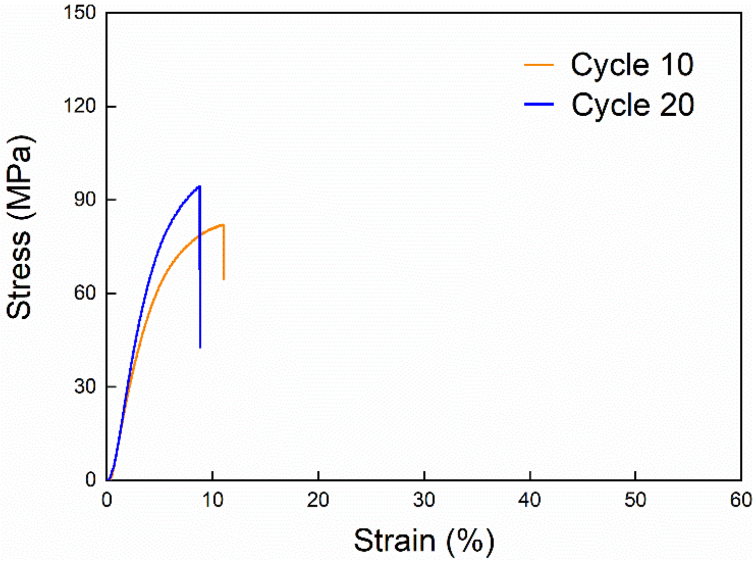

Supplementary Fig. 8.

Stress–strain curves of CPaE membranes after 10, 20 times of regeneration.

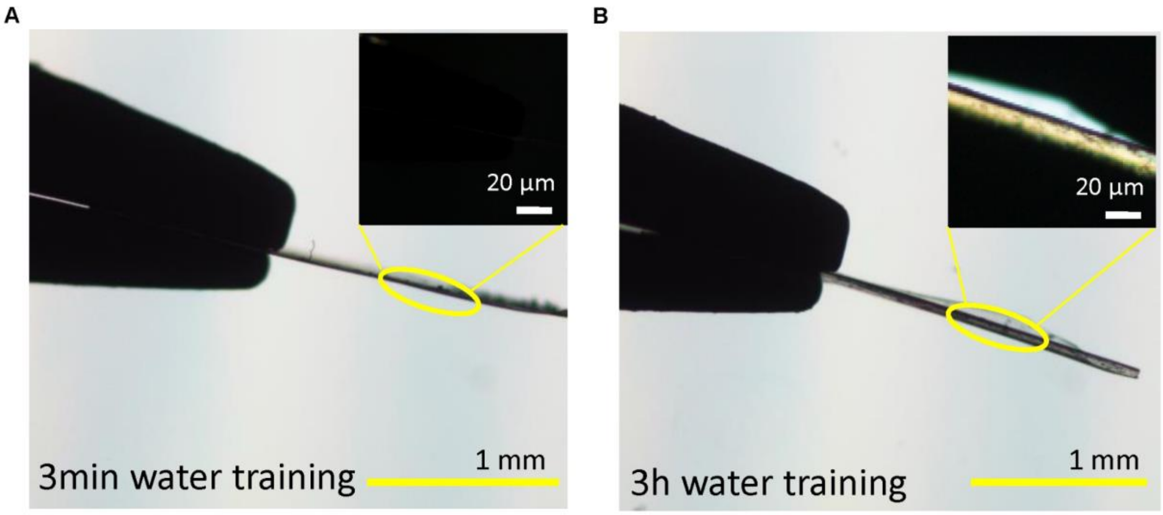

Supplementary Fig. 9.

Optical Microscopy (OM) imagines shows the cross section of air dried CPaE membrane strips being made after 3 min (a) and 3 hours (b) water training, respectively. Inset shows the enlarged Polarized Optical Microscopy (POM) imagines.

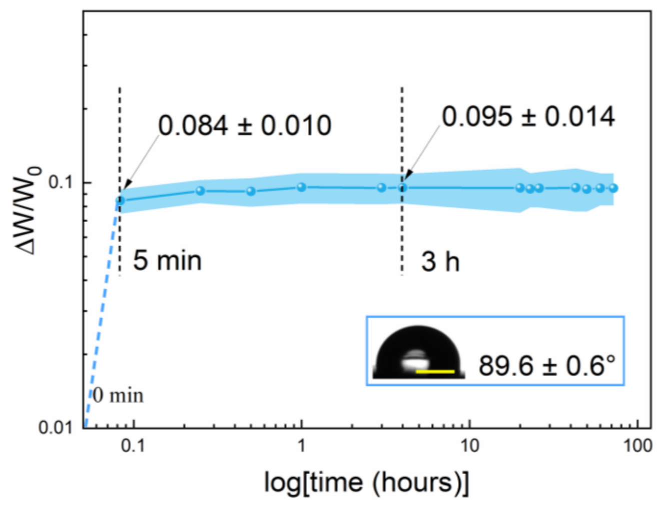

Supplementary Fig. 10

Water Sorption Dynamics in CPaE membranes. Time-dependent water sorption curve in an ambient condition (16.7-22.6 °C, 46-65% RH). Inset shows the static water contact angle on their surfaces. The scale bar is 1 mm. Error bands are SD.

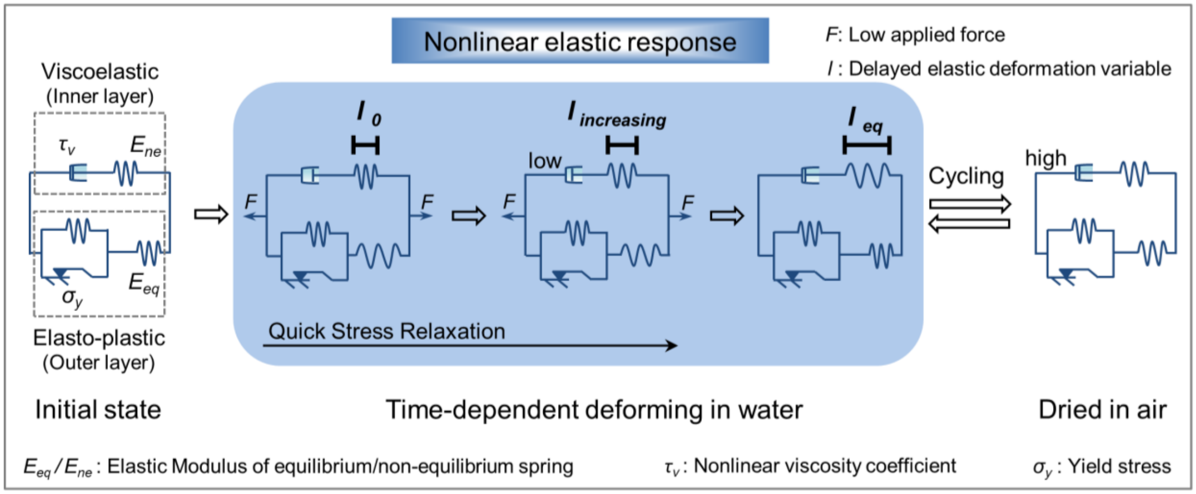

Supplementary Fig. 11.

Schematic diagram for the time-dependent nonlinear elastic response undergoing continuous force loading using modified viscoelastic-plastic model.

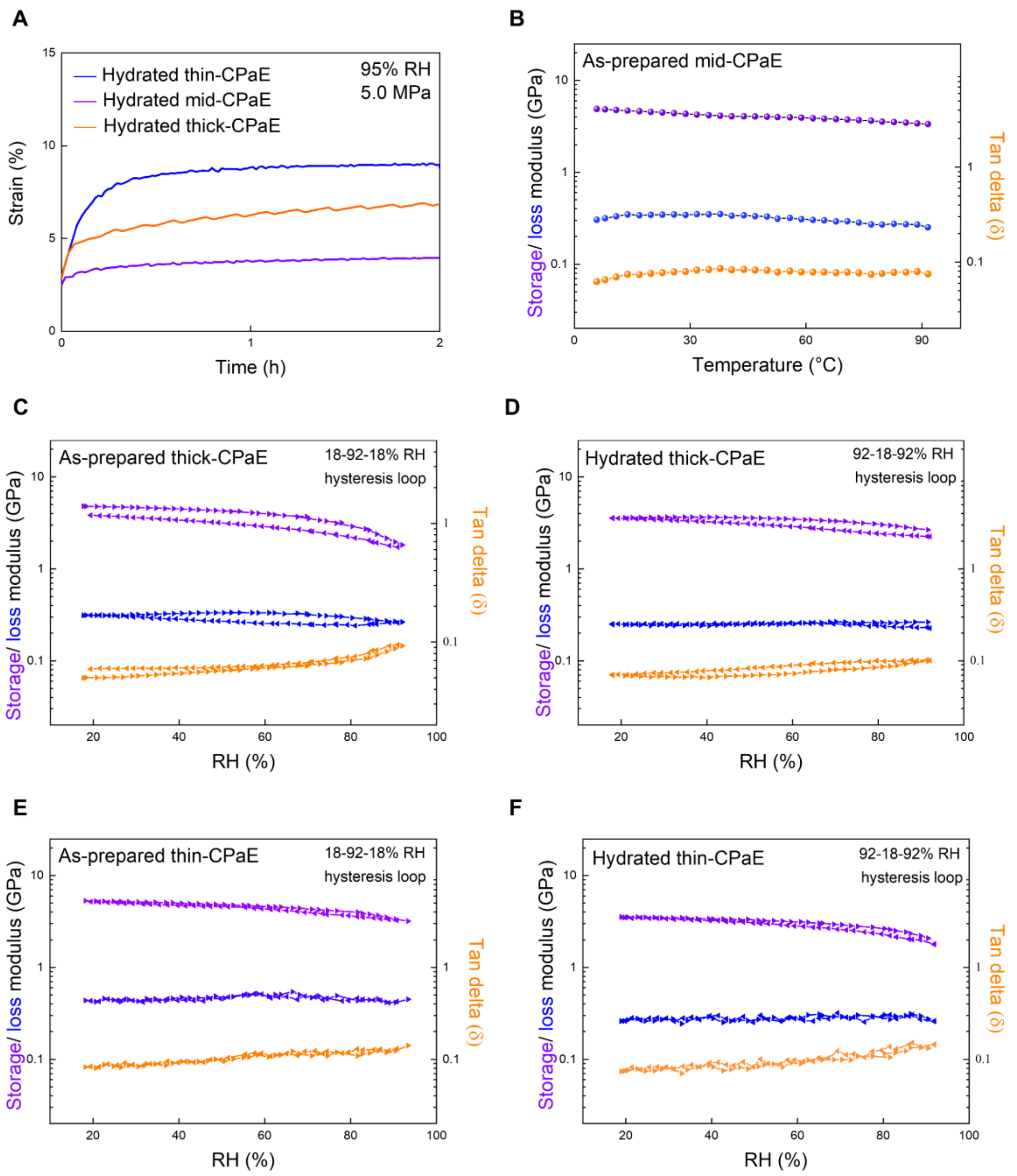

Supplementary Fig. 12.

(A) Creep behaviors of hydrated CPaE membranes loaded with constant load of 5 MPa under 25 °C, 95% RH. (B) Dynamic Mechanical Temperature Analysis (DMTA) of as-prepared mid-CPaE from 5 to 90 °C at 10 Hz. C to F, RH hysteresis loop begins at 18% RH for as-prepared (C) thick-CPaE and (E) thin-CPaE, and begins at 92% RH for the hydrated (D) thick-CPaE and (F) thin-CPaE.

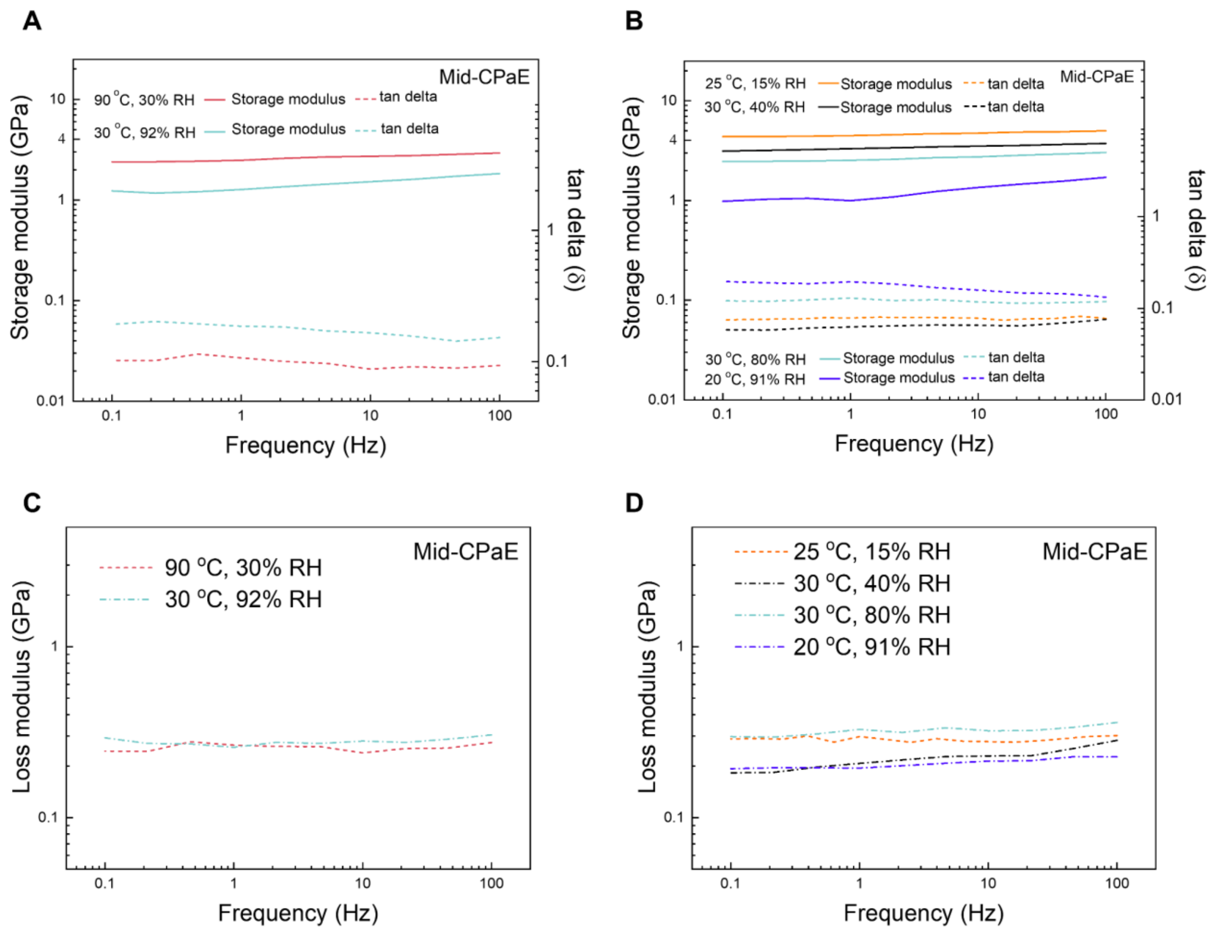

Supplementary Fig. 13.

Temperature-Frequency-Humidity sweep of mid-CPaE membranes. A to B, the storage modulus and damping factors of frequency sweep under (A) 90 °C, 30% RH and 30 °C, 92% RH, (B) 25 °C, 15% RH and 30 °C, 40% RH and 30 °C, 80% RH and 20 °C, 91% RH. C to D, the loss modulus of frequency sweep under (C) 90 °C, 30% RH and 30 °C, 92% RH, (D) 25 °C, 15% RH, 30 °C and 40% RH, 30 °C and 80% RH and 20 °C, 91% RH.

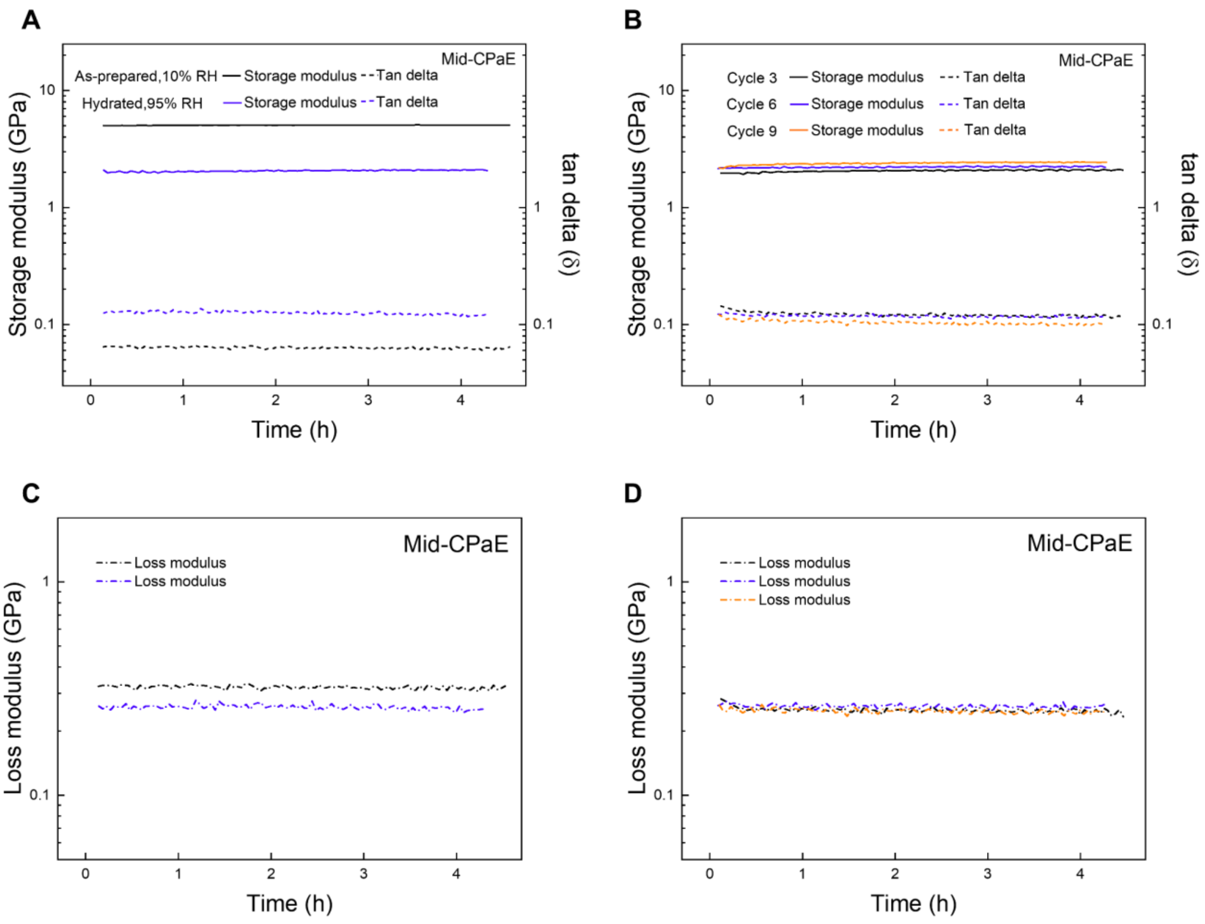

Supplementary Fig. 14.

Dynamic mechanical thermal analysis of mid-CPaE membranes. A-B, the storage modulus and damping factors of time sweep (25 °C, 10 Hz) of (A) as-prepared mid-CPaE at 10% RH, and hydrated mid-CPaE (cycle 0) at 95% RH as well as (B) those after 3, 6 and 9 times of water and air training strategies (cycle3, 6, 9). C-D, the loss modulus of time sweep (25 °C, 10 Hz) of (C) as-prepared mid-CPaE at 10% RH, and hydrated mid-CPaE (cycle 0) at 95% RH as well as (D) those after 3, 6 and 9 times of water and air training strategies (cycle3, 6, 9).

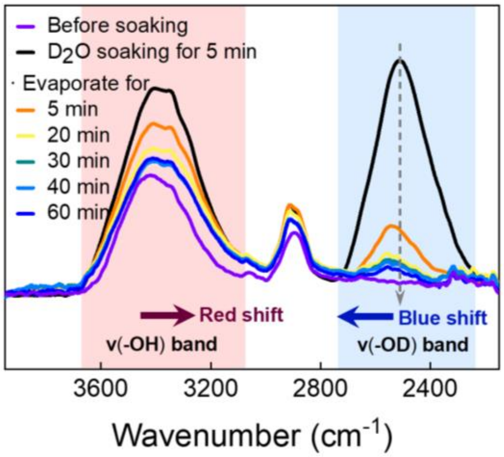

Supplementary Fig. 15.

ATR-FTIR spectra of the CPaE membranes soaked in D_2O for 5 min, and after variant time intervals of exposure to the ambient condition (25.9-26.7 ℃, 31-36% RH).

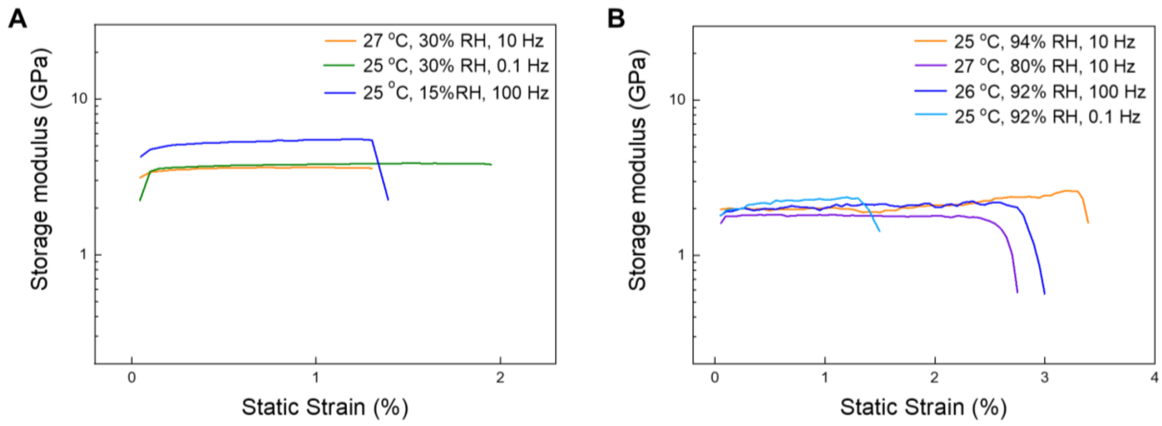

Supplementary Fig. 16.

Strain sweep of as-prepared CPaE membranes under diverse conditions including (a) high RH conditions of 80%, 92% and 94% RH and (b) low RH conditions of 15%, 30% RH representing the conditions used for DMTA measurements.

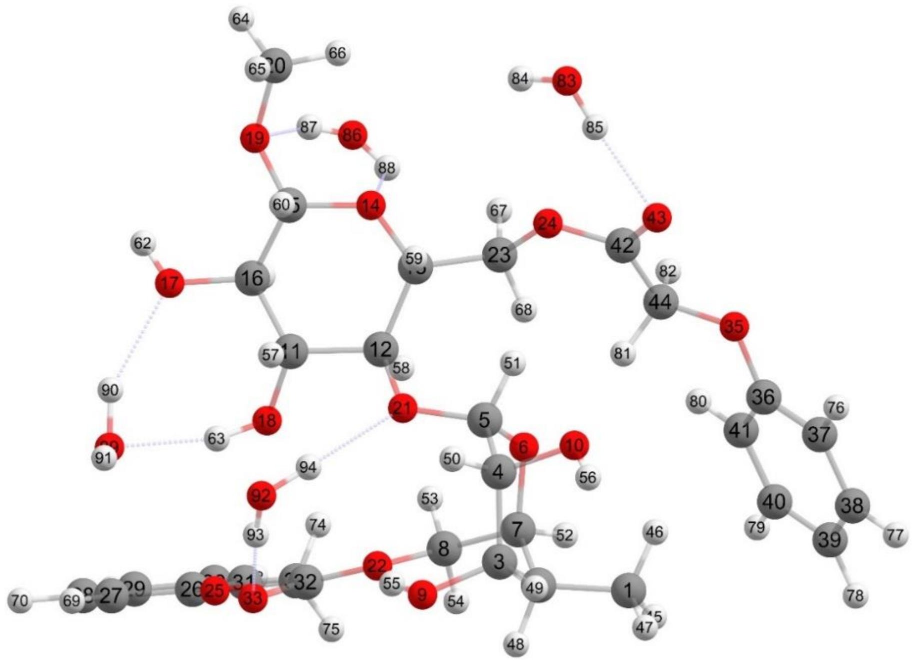

Supplementary Fig. 17.

Optimized atomic configuration of minimum point on the potential energy surface of CPaE-H_2O based on atoms in molecules theory, showing the hydrogen bonds pattern and binding sites between CPaE and H_2O. Binding energy of the complexes computed at the CCSD(T)/jul-cc-pVTZ level. The grey, white, and red colors represent carbon, hydrogen, and oxygen atoms in the model, respectively.

Supplementary Table 1.

Calculated results of electron density and H-bonds energy between CPaE and water. Binding energy of the complexes computed at the CCSD(T)/jul-cc-pVTZ level.

Location of H-bonds	Electron density at BCP (a.u.)	The fitted bond energy of H-bonds (kcal/mol)
H87……O19	0.01831	-3.34
H88……O14	0.01217	-1.97
H85……O43	0.02311	-4.41
H90……O17	0.01841	-3.37
H63……O89	0.03324	-6.67
H94……O21	0.01351	-2.27
H93……O33	0.02103	-3.95

Supplementary References

1. Heinze, T., Pohl, M., Schaller, J. & Meister, F. Novel Bulky Esters of Cellulose. *Macromolecular Bioscience* **7**, 1225-1231, doi:https://doi.org/10.1002/mabi.200700103 (2007).
2. Chen, Z., Zhang, J., Xiao, P., Tian, W. & Zhang, J. Novel Thermoplastic Cellulose Esters Containing Bulky Moieties and Soft Segments. *ACS Sustainable Chemistry & Engineering* **6**, 4931-4939, doi:10.1021/acssuschemeng.7b04466 (2018).
3. Meng, X., Matson, J. B. & Edgar, K. J. Olefin cross-metathesis as a source of polysaccharide derivatives: Cellulose ω-carboxyalkanoates. *Biomacromolecules* **15**, 177-187 (2014).
4. Vaca-Garcia, C., Borredon, M.-E. & Gaseta, A. Determination of the degree of substitution (DS) of mixed cellulose esters by elemental analysis. *Cellulose* **8**, 225-231 (2001).
5. Emamian, S., Lu, T., Kruse, H. & Emamian, H. Exploring nature and predicting strength of hydrogen bonds: A correlation analysis between atoms-in-molecules descriptors, binding energies, and energy components of symmetry-adapted perturbation theory. *Journal of computational chemistry* **40**, 2868-2881 (2019).
