# Supplementary material for: Water training initiates spatially regulated microstructures with competitive mechanics in hydroadaptive polymers
Source: Nat Commun. 2024 Jul 19;15:6093. doi: 10.1038/s41467-024-50328-7 (PMC11271527; doi:10.1038/s41467-024-50328-7)
Supplement: Supplementary file 3 — Description of Additional Supplementary Files [file 41467_2024_50328_MOESM3_ESM.pdf]

Description of Additional Supplementary Files

Supplementary Movie Legends:

Supplementary Movie 1. Robust water shapes. The water helix made from CPaE membrane strips maintain a robust shape even when a disturbance of external force is applied.

Supplementary Movie 2. Bulk mechano-responsiveness of the CPaE membrane strips to water. The air helix changing to flat shape (original shape) when immersed it in water, corresponding to a shape erasing behavior.

Supplementary Movie 3. Differential mechanoresponsiveness of the CPaE membrane strips to water. The air chair changing to pre-loaded water helix when immersed it in water, corresponding to a shape recovery behavior.

Supplementary Movie 4. Differential mechanoresponsiveness of the CPaE membrane strips to water. The air helix changing to pre-loaded water chair when immersed it in water, corresponding to a shape recovery behavior.

Supplementary Movie 5. Differential mechanoresponsiveness of the CPaE membrane strips to water. The air helix changing to pre-loaded water ring when immersed in water, corresponding to a shape recovery behavior.
